# Channel nuclear pore protein 54 directs sexual differentiation and neuronal wiring of female reproductive behaviors in *Drosophila*

**DOI:** 10.1186/s12915-021-01154-6

**Published:** 2021-10-20

**Authors:** Mohanakarthik P. Nallasivan, Irmgard U. Haussmann, Alberto Civetta, Matthias Soller

**Affiliations:** 1 School of Biosciences, College of Life and Environmental Sciences, University of Birmingham, Edgbaston, Birmingham, B15 2TT UK; 2 Department of Life Science, School of Health Sciences, Birmingham City University, Birmingham, B15 3TN UK; 3 Department of Biology, University of Winnipeg, Winnipeg, MB R3B 2E9 Canada; 4 Birmingham Centre for Genome Biology, University of Birmingham, Edgbaston, Birmingham, B15 2TT UK

**Keywords:** *Nup54*, Nuclear pore complex (NPC), Sexual differentiation, Neuronal wiring, Post-mating behaviors, *pickpocket* (*ppk*) neurons

## Abstract

**Background:**

Female reproductive behaviors and physiology change profoundly after mating. The control of pregnancy-associated changes in physiology and behaviors are largely hard-wired into the brain to guarantee reproductive success, yet the gene expression programs that direct neuronal differentiation and circuit wiring at the end of the sex determination pathway in response to mating are largely unknown. In *Drosophila*, the post-mating response induced by male-derived sex-peptide in females is a well-established model to elucidate how complex innate behaviors are hard-wired into the brain. Here, we use a genetic approach to further characterize the molecular and cellular architecture of the sex-peptide response in *Drosophila* females.

**Results:**

Screening for mutations that affect the sensitivity to sex-peptide, we identified the channel nuclear pore protein *Nup54* gene as an essential component for mediating the sex-peptide response, with viable mutant alleles leading to the inability of laying eggs and reducing receptivity upon sex-peptide exposure. *Nup54* directs correct wiring of eight adult brain neurons that express *pickpocket* and are required for egg-laying, while additional channel Nups also mediate sexual differentiation. Consistent with links of Nups to speciation, the *Nup54* promoter is a hot spot for rapid evolution and promoter variants alter nucleo-cytoplasmic shuttling.

**Conclusions:**

These results implicate nuclear pore functionality to neuronal wiring underlying the sex-peptide response and sexual differentiation as a response to sexual conflict arising from male-derived sex-peptide to direct the female post-mating response.

**Supplementary Information:**

The online version contains supplementary material available at 10.1186/s12915-021-01154-6.

## Background

In most insects, male-derived substances transferred during mating direct female physiology and post-mating behaviors [1, 2]. *Drosophila* females display a repertoire of sex-specific behaviors after mating including reduced receptivity (readiness to mate) and increased egg-laying [3]. The main trigger of these post-mating behaviors is male-derived sex-peptide (SP). SP is a 36 amino acid peptide that is transferred during mating to the female. Besides reducing receptivity and increasing egg-laying, SP induces a number of other behavioral and physiological changes including increased egg production, feeding, a change in food choice, sleep, memory, constipation, and stimulation of the immune system [4–11]. In addition, SP binds to the sperm and acts as a sperm sensor, is required for the release of the stored sperm, and imposes costs of mating [12–14].

Many of the phenotypic effects of SP on female physiology are known, but the central aspects of the neuronal circuitry governing the regulation of the main post-mating behaviors like reduced receptivity and increased oviposition are unclear. First insights came from the analysis of mutant alleles of the *egghead* gene, encoding a 1,4 mannosyltransferase involved in glycosphingolipid biosynthesis, that are insensitive to SP and required for neuronal wiring [15, 16]. In these mutants, neuronal connections from the ventral nerve cord to the central brain show defects resulting in an egg retainer phenotype and non-responsiveness to SP in reducing receptivity. This suggests that receptor signaling is disconnected from the motor output programs.

Other attempts to map the circuitry mediating the post-mating response used expression of membrane-tethered SP or RNAi knockdown of a receptor for SP, SPR, in distinct neuronal expression patterns [17–21]. These screens identified *pickpocket* (*ppk*), *fruitless* (*fru*), and *dsx* expressing neurons mediating the post-mating switch via expression of membrane-tethered SP in multiple pathways [21].

The ability of males to manipulate post-mating responses of females, for example, by SP in *Drosophila,* can promote sexual conflict and trigger an arms race between the sexes driven by the female escape of male manipulation [22–24]. This arms race drives rapid evolution and can fuel speciation. Few genes have been linked to speciation by mapping inviability or sterility in hybrids of close relatives. Among them are the two essential nuclear pore proteins, *Nup96* and *Nup160*, that are part of the outer ring of the pore and interact with each other and that can trigger inviability in species hybrids through adaptive divergence [25–28]. The megadalton nuclear pore complex constitutes a bidirectional gateway connecting the nucleus and cytoplasm to control the transport of all macromolecules [29], but how nuclear pore proteins can drive speciation is unknown. Here, we show that *Nup54* directs nucleo-cytoplasmic shuttling important for sexual differentiation including wiring of the neurons required for the female post-mating response to SP. Moreover, the *Nup54* promoter is a hot spot for rapid diversification consistent with a role in sexual conflict-driven speciation.

## Results

### *Nup54* mutant females retain eggs and are insensitive to sex-peptide for reducing receptivity

**Fig. 1:**
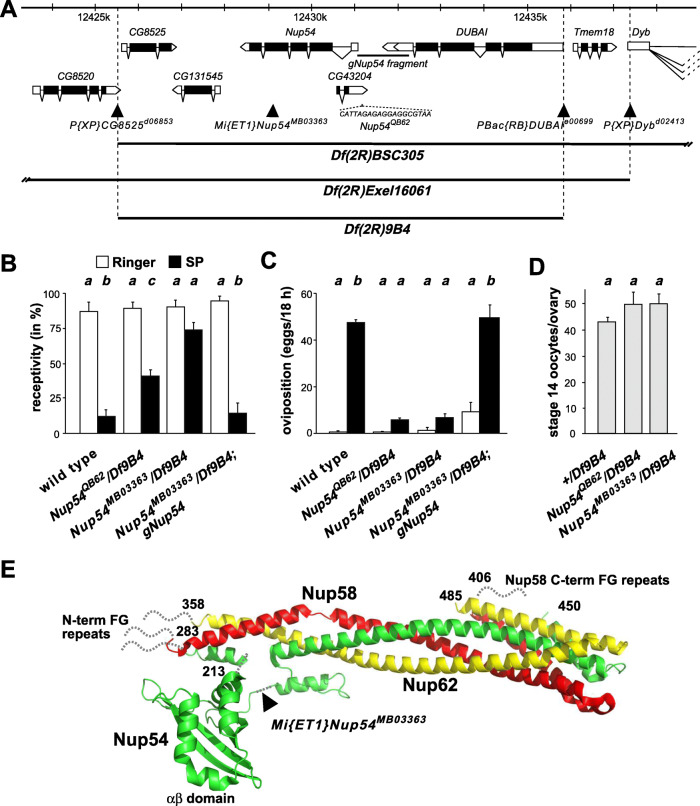
Mapping of sex-peptide insensitive EMS allele *QB62* to *Nup54*. **A** Schematic of the *Nup54* chromosomal region depicting gene models and chromosomal deficiencies used below the chromosomal nucleotide positions. Coding parts are shown as black and non-coding parts as white boxes. Transposon insertions are show as triangles. The sequence of the deletion in *Nup54*^*QB62*^, and the promoter fragment used for genomic rescue construct *gNup54* are shown below the gene model. **B** Receptivity of wild type and transheterozygous *Nup54*^*QB62*^*/Df(2R)9B4, Nup54*^*MB03363*^*/Df(2R)9B4*, and *Nup54*^*MB03363*^*/Df(2R)9B4; gNup54* females after sex-peptide (SP, black) or Ringer’s (R, white) injection measured by counting mating females in a 1 h time period 3 h after SP or R injection, respectively. The means with the standard error for three or four experiments with 18–23 females each are shown, and statistically significant differences from ANOVA post hoc pairwise comparisons are indicated by different letters (**a**, **b** *P*≤0.05, **c** ns). **C** Oviposition of wild-type and transheterozygous *Nup54*^*QB62*^*/Df(2R)9B4*, *Nup54*^*MB03363*^*/Df(2R)9B4*, and *Nup54*^*MB03363*^*/Df(2R)9B4*; *gNup54* females after sex-peptide (SP, black) or Ringer’s (R, white) injection shown as means of eggs laid in 18 h with the standard error for 10 females each, respectively, and statistically significant differences from ANOVA post hoc pairwise comparisons are indicated by different letters (*P*≤0.0001). **D** Number of stage 14 oocytes present in ovaries of sexually mature virgin wild type and transheterozygous *Nup54*^*QB62*^*/Df(2R)9B4* and *Nup54*^*MB03363*^*/Df(2R)9B4* females shown as means with the standard error for seven ovaries. **E** Ribbon diagram of the structure of the channel Nup complex consisting of Nup54, Nup58, and Nup62 with the position of the *Mi{ET1}Nup54*^*MB03363*^ transposon insertion indicated leading to a partially functional truncated protein. The data underlying the presented graphs are in Additional file 6

If the SP response is disrupted to render females insensitive to SP, they retain eggs and remate, suggesting a common part in the signaling pathway leading to egg-laying and refractoriness to remate upon SP exposure [15]. To identify genes involved in specifying the SP response, we identified lines where females have normal oogenesis, but do not lay eggs. We then tested virgin females of these lines after SP injection for their ability to reduce receptivity [15]. Using this approach, we identified a homozygous viable EMS-induced line, *QB62* [30] that did not reduce receptivity upon injection of SP (Fig. 1A–D). We mapped this allele by meiotic recombination based on the egg retention phenotype to 2–69.9, corresponding approximatively to chromosome position 50C. Using overlapping deficiencies this allele could then be mapped to chromosome section 49A4 by deficiencies *Df(2R)BSC305* and *Df(2R)Exel16061* (Fig. 1A). To further restrict the number of genes in this chromosomal area, we generated a smaller deficiency, *Df(2R)9B4*, by FRT-mediated recombination between transposons *P{XP}CG8525*^*d06853*^ and *PBac{RB}DUBAI*^*e00699*^ (Fig. 1A).

We then identified a GFP-marked *Mi{ET1}*transposon insert in the *Nup54* gene, *Nup54*^*MB03363*^, that was allelic to *QB62* with respect to both egg retention and reduction of receptivity after SP injection (Fig. 1B–D). Nup54 localizes to the transport channel of the megadalton nuclear pore complex that constitutes a bidirectional gateway connecting the nucleus and cytoplasm to control the transport of all macromolecules [29]. The *Nup54*^*MB03363*^ transposon insert leads to a truncation of the Nup54 ORF and removes the C-terminal alpha-helical domain that connects Nup54 with Nup58, but leaves the core part containing the FG repeats composing the inner nuclear channel and the interaction of Nup54 with Nup62 via the alpha/beta helical region intact (Fig. 1E) [29]. Sequencing of the *Nup54*^*QB62*^ allele identified a small deletion (CATTAGAGAGGAGGCGTAA) in the promoter region 186 nt upstream of the first transcribed nucleotide. These two alleles represent hypomorphic mutations.

To further confirm that egg retention and SP insensitivity in receptivity maps to the *Nup54* gene, we used a genomic rescue construct. For this construct, a fragment upstream of the transcription start site (indicated by a line in Fig. 1A) was fused to the transcribed part, where the first intron containing the overlapping gene *CG43204* (*gNup54*) was deleted. The presence of this construct as in *Nup54*^*MB03363*^*/Df(2R)9B4; gNup54* fully rescued the egg retention and SP insensitivity in receptivity (Fig. 1B, C) and also rescued the lethality of *Df(2R)9B4* indicating that *Nup54* is essential and the only lethal gene in *Df(2R)9B4*.

### *Nup54* is required in the development for establishing the post-mating response

The SP-induced change in post-mating behaviors is mediated via neuronal signaling [3]. To identify the temporal and spatial requirement of *Nup54* during the development of the nervous system, we aimed to rescue the egg retainer and SP insensitivity phenotype in receptivity in *Nup54* mutant females by expressing *UASNup54* in a *Nup54*^*MB03363*^*/Df(2R)9B4* background with various *GAL4* drivers. Global expression starting early in development with *tubGAL4* in *Nup54*^*MB03363*^*/Df(2R)9B4* completely rescued these phenotypes after injection of SP (Additional file 1 A and B). When we then expressed *UASNup54* in all differentiated neurons with *elavGAL4*^*C155*^, refractoriness to remate was not rescued and oviposition increased only marginally in the presence of SP in *Nup54*^*MB03363*^*/Df(2R)9B4* (Additional file 1 A and B). This result indicates that *elavGAL4* expresses too late in development to rescue insensitivity to SP to revert irreversible aberrant neuronal development (see below). Compared to the expression of endogenous *Nup54*, *GAL4/UAS*-mediated expression is delayed because GAL4 needs to be first made to activate transcription from *UAS*. As a consequence, some gene knock-outs such as *elav* cannot be rescued for this reason [31, 32], but could also be a result of insufficient driver strength in some cells. Expression of *UASNup54* in glial cells with *repoGAL4* in *Nup54*^*MB03363*^*/Df(2R)9B4* rescued receptivity, but not oviposition after SP injection, indicating that the development of the neuronal circuitry mediating the post-mating response is supported by glial cells (Additional file 1 A and B). Since both *elavGal4* and *repoGal4* did not fully rescue, it is likely that Nup54 is required in both neurons and glia for the rescue of SP-insensitivity.

We then expressed *UASNup54* in those subsets of neurons that can induce the SP response from the expression of membrane-tethered SP using *ppkGAL4*, *fruGAL4*, and *dsxGAL4* in *Nup54*^*MB03363*^*/Df(2R)9B4* after SP injection (Additional file 1 A and B). These experiments revealed that *fruGAL4* and *dsxGAL4* can rescue refractoriness to remate induced by SP, but only *dsxGAL4* can marginally rescue egg-laying. These results indicate that irreversible aberrant neuronal development has taken place before these *GAL4* drivers express or that other neurons directing the post-mating response are involved (Additional file 1 A and B).

Taken together, these data show that Nup54 is required early in development before neurons are fully differentiated and in glial cells to specify the neuronal circuits required for the post-mating response as the mutant phenotype can only be rescued by early expressing *GAL4* lines. Moreover, our data confirm that the neuronal circuits for egg-laying and receptivity are not identical [21].

### *Nup54* is required for neuronal wiring of *pickpocket* neurons

**Fig. 2:**
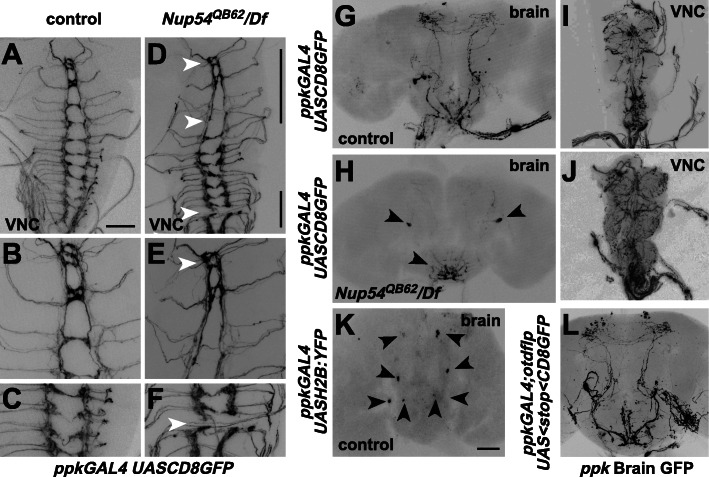
*Nup54* is required to establish neuronal projections of *pickpocket* neurons. A–F Representative larval ventral nerve cord expressing CD8::GFP from *UAS* by *ppkGAL4* in wild type (**A**–**C**) and *Nup54*^*QB62*^*/Df(2R)9B4* (**D**–**F**)*.* Magnified corresponding areas indicated in **D** are shown for wild-type (**B** and **C**) and *Nup54*^*QB62*^*/Df(2R)9B4* (**E** and **F**). Arrowheads indicate missing commissures or connectives. **G–J** Representative adult brain and ventral nerve cord (VNC) expressing CD8::GFP from *UAS* by *ppkGAL4* in wild type (**G** and **I**) and *Nup54*^*QB62*^*/Df(2R)9B4* (**H** and **J**)*.* Arrowheads point towards cell bodies of *ppk* expressing neurons with absent neuronal arborizations. **K** Representative adult brain expressing histone2B::YFP from *UAS* by *ppkGAL4* in wild-type. Arrowheads point towards cell bodies of the four paired *ppk* expressing neurons in the adult brain. The scale bar in **A** is 50 μm and in **K** is 100 μm. **L** Representative adult brain expressing CD8::GFP only in head *ppk* neurons by the intersection of *ppkGAL4/UAS FRTstopFRT CD8GFP* expression with *otdflp*, which expresses only in the brain

To evaluate whether *Nup54*^*QB62*^*/Df(2R)9B4* mutant females had any gross morphological changes in the brain, we analyzed neuronal projections of *ppk* and *dsx* neurons as these neurons are directly relevant to female sexual behaviors [17–19, 21] (Fig. 2 and Additional file 2). Moreover, since *ppkGAL4* and *dsxGAL4* mark only few neurons with distinct axonal tracts as shown with *UASCD8GFP* expression, they are well suited to evaluate the morphology of relevant neurons (Fig. 2G, H, Additional file 2 A and B).

For *dsx* neuronal projections in *Nup54*^*QB62*^*/Df(2R)9B4* females, we did not detect alterations in the brain (*n*=6), the ventral nerve cord (*n*=6), and the genital tract (*n*=6) compared to wild type (Additional file 2 A-F). In contrast, projections of *ppk* neurons in the larval brain of *Nup54*^*QB62*^/*Df(2R)9B4* showed severe disruption with connectives and/or commissures in 50% (*n*=20) of the brains, and moderate and mild aberrations in half of the remaining brains each compared to wild type (Fig. 2A–F). In the adult brain, projections in the central brain were severely aberrant in 89% of *Nup54*^*QB62*^/*Df(2R)9B4* females (*n*=9) and mildly aberrant in the remaining brains compared to control females (*n*=10, Fig. 2G, H), while no projection differences were obvious in the ventral nerve cord (*n*=5, Fig. 2I, J). A closer examination of *ppkGAL4* expression with nuclear *UAShistone2B::YFP*, however, revealed four paired neurons that are consistently present in the adult female brains (*n*=5, Fig. 2K) [33].

**Fig. 3:**
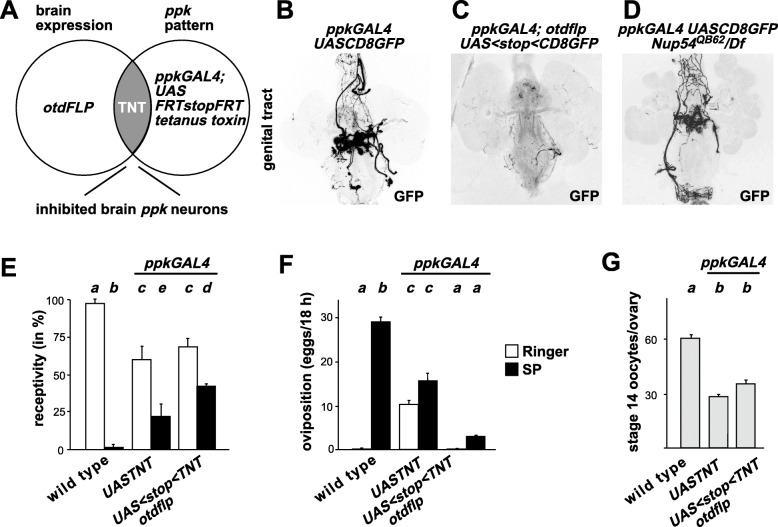
Brain *pickpocket* neurons are part of the circuit directing egg-laying. **A** Schematic of the intersectional gene expression approach to direct expression of tetanus toxin (TNT) to brain *ppk* expressing neurons using the brain-expressed *otdflp* and *UAS FRTGFPstopFRTTNT*. **B–D** Representative genital tracts of indicated genotypes showing CD8GFP expression in *ppk* neurons. Note that intersectional directed expression of *ppk* to the head in *ppkGAL4*/*UAS FRTGFPstopFRTCD8GFP*; *otdflp* (**C**) removes expression in genital tract neurons. **E** Receptivity of control and females expressing TNT in all (*ppkGAL4*) or only brain *ppk* neurons (*ppkGAL4; UAS stop TNT/otdflp*) after sex-peptide (SP, black) or Ringer’s (R, white) injection measured by counting mating females in a 1-h time period 3 h after SP or R injection, respectively. The means with the standard error for three experiments with 10–24 females each are shown, and statistically significant differences from ANOVA post hoc pairwise comparisons are indicated by different letters (**a**–**d**: *P*≤0.05, **e** ns). **F** Oviposition of control and females expressing TNT in all (*ppkGAL4*) or only brain *ppk* neurons (*ppkGAL4; UAS stop TNT/otdflp*) after sex-peptide (SP, black) or Ringer’s (R, white) injection shown as means of eggs laid in 18 h with the standard error for 6–10 females, respectively. Statistically significant differences from ANOVA post hoc pairwise comparisons are indicated by different letters (*P*≤0.001). **G** Number of stage 14 oocytes present in ovaries of control and females expressing TNT in all (*ppkGAL4*) or only brain *ppk* neurons (*ppkGAL4; UAS stop TNT/otdflp*) shown as means with the standard error for six ovaries. Statistically significant differences from ANOVA post hoc pairwise comparisons are indicated by different letters (*P*≤0.001). The data underlying the presented graphs are in Additional file 6

Most prominently, *ppk* expresses in sensory neurons projecting into the brain [17–19], but their contribution to arborizations of *ppk* neurons in the brain has not been established. Since ascending neurons from the genital tract and the ventral nerve cord project to the brain, we used *flipase (flp)* expression under an *orthodenticle* (*otd*) promoter [34], which is restricted to the brain to determine whether the arborizations in the brain are from these four *ppk* neuron-pairs or result from projections from neurons in the genital tract. No difference in the projections in the central brain was observed when *ppk* expression was restricted to the brain (*n*=4, Fig. 2L) with an intersectional approach (Fig. 3A). Accordingly, GFP expression was absent in genital tract *ppk* neurons in these females (*n*=4) as *otd-flp* is only expressed in the head and therefore does not remove the stop cassette in *UASCD8FRTstopFRTGFP* in the genital tract (Fig. 3B, C). In *Nup54*^*QB62*^/*Df(2R)9B4* genital tracts, *ppk* neurons were present and showed no major arborization defects or disruption in the projections (*n*=4, Fig. 3D).

### *ppk* neurons in the brain direct egg-laying

Since expression of membrane-tethered SP with *ppkGAL4* induces a post-mating response, we wanted to test whether *ppk* expressing neurons in the brain are involved in the post-mating response. For this experiment, we used the same intersectional gene expression approach using *otd-flp* to specifically inhibit neuronal transmission in brain *ppk* neurons by expression of tetanus toxin (TNT) (Fig. 3A). To achieve restricted expression of TNT in *ppk* brain neurons, we crossed *otd-flp* flies with *ppkGAL4; UAS FRTGFPstopFRT TNT* flies and analyzed the female progeny for the post-mating response.

These experiments revealed that females with inhibited *ppk* neurons in the brain or with all *ppk* neurons inhibited displayed a partial response to SP as they did not fully reduce receptivity (Fig. 3E). Females with inhibited *ppk* neurons in the brain did not lay the eggs stored in the ovaries after injection of SP in contrast to inhibition of all *ppk* neurons which resulted in increased egg-laying in virgins and SP does not further enhance egg-laying (Fig. 3G, F). Control females of *UAS TNT* without a *GAL4* driver showed a normal response to SP [15].

Taken together, these results demonstrate an essential role for *ppk* neurons in the brain in inducing egg-laying, and a partial requirement for reducing receptivity in response to SP, but also that *ppk* neurons outside the brain are inhibitory with regard to egg-laying as reported previously [17–19].

### Channel *Nups* have a role in sex determination

To further examine the role of *Nup54* in the post-mating response, we used *UASRNAi* knock-down of *Nup54* ubiquitously with *tubGAL4*, in all neurons using *elavGAL4*^*C155*^, or in subsets of neurons using *ppkGAL4*, *fruGAL4*, and *dsxGAL4*. RNAi knock-down of *Nup54* with *tubGAL4* is lethal, while *elavGAL4* ^*C155*^, *ppkGAL4*, and *fruGAL4* did not affect receptivity, or egg-laying after SP injection (Additional file 3 A and B). This result is not unexpected since Nup54 is required early in neuronal development to rescue SP insensitivity in *Nup54* mutant females, as shown by the lack of rescue from *GAL4* lines expressing a rescue construct late in development, but alternatively, RNAi knock-down could insufficiently reduce *Nup54* expression. In contrast, RNAi knock-down of *Nup54* with *dsxGAL4*, oviposition was not induced upon SP injection while receptivity was reduced normally (Additional file 3 A and B).

We further noticed that these *dsx Nup54* RNAi females laid eggs that did not develop. Moreover, male survival was low (20%, *n*=107, *P{TRIP.HMC04733}*) or males were completely absent (*n*=107, *P{GD14041}v42153; P{GD14041}v42154*). In the progeny of the mild hypomorphic *Nup54*^*QB62*^ and *Nup54*^*MB03363*^ alleles crossed to *Df(2R)9B4*, no bias in male survival was detected (98% males, *n*=194 and 92% males, *n*=142, respectively).

Since Nup54 forms a complex with two other channel Nups, Nup58 and Nup62, we wanted to test by RNAi whether they have roles in specifying the post-mating response and/or in sexual differentiation. RNAi knockdown of *Nup58* (*P{TRIP.HMC05104}*) with *dsxGAL4* resulted in lethality with only few very weak female escapers. These females, however, were sterile due to the lack of ovaries and deformed genitals, but looked otherwise normal. Similar results were obtained by RNAi knock-down of *Nup62* with *dsxGAL4* resulting in male lethality (9%, *n*=81 and 2%, *n*=45 escapers with *P{TRIP.GLV21060}* and *P{TRIP.HMC03668}*, respectively) and females without ovaries (100%, *n*=74 and 43%, *n*=35 with *P{TRIP.GLV21060}* and *P{TRIP.HMC03668}*, respectively). In addition, these females also had deformed genitals and males displayed underdeveloped sex combs indicating a role for channel Nups in sexual differentiation (Additional file 3 C-F). Consistent with a regulatory role in development, the channel *Nups* display dynamic expression during development and in different tissues (Additional file 4 A and B) [35, 36].

### The role of indels during the evolution of Nup54 function

Changes in the protein sequence of Nups have been attributed a role in speciation as an adaptation to new situations drives the evolution of *Nup96* and *Nup160* genes by leading to hybrid incompatibility [25–27]. Therefore, we tested whether the Nup54 coding region is under selection. We used the combined analysis of polymorphism within *D. melanogaster* and divergence between *D. melanogaster* and *D. simulans* within the coding region of *Nup54* to test for deviations from expectations under the neutral model of molecular evolution. We found no deviation from the null neutral model hypothesis, as the ratio of nonsynonymous to synonymous polymorphisms did not differ significantly from the ratio of nonsynonymous to synonymous fixed differences between species (MK test: *χ*^2^= 0.09; *P*= 0.923). The test was also non-significant when we partition the analysis by exons.

When we compared the sequences in more distantly related species, we noticed that a stretch of amino acids increased in length inserted in the FG repeat region constituting the transport channel (Fig 1E and Additional file 5 A). Indels (insertions/deletions) are a common form of genetic variation that can affect gene function and their pattern of evolution under selective forces [37]. We therefore hypothesized that this indel could alter the function of *Nup54* in specifying the post-mating response.

To test this hypothesis, we replaced the FG-repeat region from *D. melanogaster* with the region from *D. elegans* in the genomic rescue construct *gNup54ele* and tested its capacity to rescue viability of *Df(2R)9B4* and the post-mating response defects. Both the *D. melanogaster gNup54* and the chimeric *D. elegans* construct *gNup54ele* rescued viability of *Df(2R)9B4* to 91% (*n*=242) and 95% (*n*=381) without sex bias, respectively. After injection of SP, females rescued with the *gNup54* or the *gNup54ele* construct showed normal post-mating responses in receptivity and oviposition arguing that this indel is not the source for an altered post-mating response (Additional file 5 B and C).

**Fig. 4:**
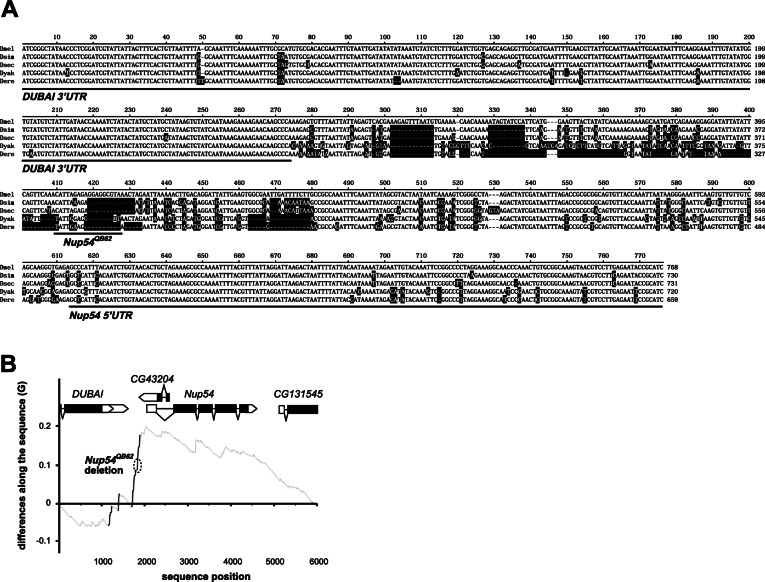
*Nup54* promoter region is diverged in closely related species. **A** Sequence alignment of the *Nup54* promoter region from closely related species. Nucleic acids deviating from *D. melanogaster* are indicated in black. Transcribed parts of the *DUBAI* 3′UTR and the *Nup54* 5′UTR, as well as the *Nup54*^*QB62*^ allele are underlined. **B** Plot of cumulative differences along the sequence (G) between the relative occurrences of nucleotide changes and their position from the alignment of the gene region around *Nup54* between *D. melanogaster* and *D. simulans*. Positions in the alignment with significant stretches of substitutions (hotspots) are identified by solid lines. The deletion in the *Nup54*^*QB62*^ allele is indicated by a circle with a dashed line. The data underlying the presented graphs are in Additional file 6

Since the coding region of Nup54 is not under selection, we next examined the *Nup54* promoter region. Sequence analysis in this region identified rapid evolution in four hotspots for substitutions between closely related species, all within 1 kb upstream of the *Nup54* transcription start site (Fig. 4A). Interestingly, one substitutional hotspot (−359 to −295) includes an indel that overlaps with the location of the characterized *Nup54*^*QB62*^ regulatory allele (Fig. 4B). The deletion in the *Nup54*^*QB62*^ allele in fact is to a large extent a wild-type condition in sister species (Fig. 4A).

**Fig. 5:**
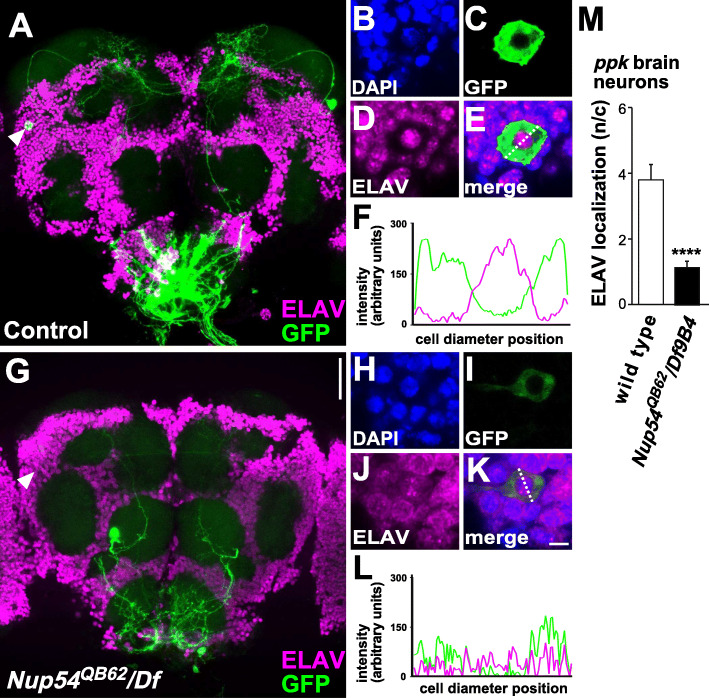
Nucleo-cytoplasmic shuttling is altered in *Nup54*^*QB62*^. **A**–**M** Cellular localization of nucleo-cytoplasmic shuttling RNA-binding protein ELAV (magenta) in representative control (**A**–**F**) and *Nup54*^*QB62*^*/Df(2R)9B4* (**G**–**L**) expressing *UASCD8GFP* from *ppkGAL4* (green). Arrowheads in **A** and **G** indicate the *ppk* expressing neuron in higher magnification for the control (**B**–**E**) and *Nup54*^*QB62*^*/Df(2R)9B4* (**G**–**K**) and the line in **E** and **K** indicate the position where signal intensities (arbitrary units) were measured for GFP (green) and ELAV (magenta) plotted in **F** and **L** of a representative example (*n*=8). **M** Quantification of ELAV signals in nuclear and cytoplasmic cellular compartments in wild type and *Nup54*^*QB62*^*/Df(2R)9B4* plotted as mean with the standard error of the ratio of the nuclear to the cytoplasmic signal (*P*≤0.0001). The scale bar in **G** is 50 μm and in **K** is 1 μm. The data underlying the presented graphs are in Additional file 6

To evaluate whether the *QB62* allele of *Nup54* affects nucleo-cytoplasmic shuttling, we stained brains for ELAV, an RNA-binding protein that shuttles between the nucleus and cytoplasm [32]. While in the brains of wild-type flies, ELAV is mostly nuclear, it is more cytoplasmic in *Nup54*^*QB62*^*/Df(2R)9B4* brains (Fig. 5A–L). Analysis of ELAV’s cellular distribution specifically in these central brain *ppk*-expressing neurons with aberrant projections revealed that ELAV was distributed equally in nucleus and cytoplasm (Fig. 5H–L) compared to strong nuclear localization in wild-type *ppk*-expressing neurons, but whether nucleo-cytoplasmic shuttling is altered in species with QB62-like promoters remains to be tested (Fig. 5B–F).

## Discussion

Here, we show that Nup54, which is part of the central transport channel of the nuclear pore, exerts a role in specifying the neuronal circuits involved in mediating the SP-induced post-mating switch. Viable alleles of these genes display an egg retainer phenotype and are insensitive to SP with regard to reducing receptivity. *Nup54* is required for wiring of *ppk* expressing neurons in the brain that constitute part of the circuitry required for egg-laying. The role of *Nup54* and the other two-channel *Nups*, *Nup58* and *Nup62*, in sexual differentiation, however, seems to be more fundamental as reducing their expression levels impacts on general sexual differentiation of external and internal sexual features, such as female genitals and ovaries.

### Roles for Nups in development

A key role for Nups in sexual differentiation seems rather unexpected as the function of channel Nups in directing constitutive export of mRNAs and proteins from the nucleus to the cytoplasm has been viewed as static for a long time. However, a number of recent studies have shown developmental and neuronal roles for the nuclear pore and nucleocytoplasmic transport reflected in a number of human diseases including neurodegeneration associated with specific Nups [38–40]. A central aspect of these more profound roles for Nups lay in their varying expression levels between different cell types, tissues, and in development. Nup210’s key role in muscle and neuronal differentiation is associated with tissue-specific expression [41]. Likewise, high expression levels of Nup153 are critical for the maintenance of ESC pluripotency as reducing levels results in neuronal differentiation [42]. Also, channel Nup62 shows increased expression in various epithelia and is required for proliferation [43]. In *Drosophila*, channel Nups 54, 58, and 62 show increased expression in the larval brain and adult gonads, but a more specific role is indicated in sexual development in *Drosophila* from broad RNAi knock-down with *dsxGAL*, recapitulating that *dsx* expression cell-autonomously directs sex determination [44, 45]. Our findings that channel Nups have a role in sexual differentiation add to this view that the nuclear pore has key roles in differentiation and likely also neuronal function.

We observed that RNAi of *Nup54* and *Nup62* results in male lethality. We did not see a sex bias in the mild hypomorphic alleles of *Nup54* likely because they retain considerable functionality. In contrast, RNAi of *Nup58* resulted in general lethality. Differences among channel Nups have also been seen in piRNA biogenesis of the *flamenco* locus [46]. Even though channel Nups form a complex, they could have individual roles in nucleo-cytoplasmic shuttling pathways, but alternatively, phenotypic differences could also be a result of the different strengths of the RNAi lines. Saturation screens for male-lethality revealed that all identified male-lethal mutants mapped to genes regulating dosage compensation [32, 44, 45], but alternative scenarios are possible involving a stronger role of channel Nups in male than female sexual differentiation. Moreover, MSL-2 expression in females is further inhibited by nuclear retention of *msl-2* mRNA by SXL and HOW RNA-binding proteins [47], but whether *Nup54* and *Nup62* have indeed a role in this process requires further validation.

### Specification of neuronal circuits for receptivity and egg-laying are separable

It has previously been argued that the neurons co-expressing *fru*, *dsx*, and *ppk* in the genital tract sense SP and direct the SP-induced post-mating response [17–19]. However, our data from expressing a *Nup54* rescue construct in various cell types suggests a more complex picture including separation of the paths leading to reduced receptivity and increased egg-laying. Here, the expression of *Nup54* in *fru* and *dsx* neurons, but not *ppk* neurons rescues receptivity while egg-laying is not rescued in all three expression patterns. Although this effect could be explained by different expression levels of *fru*, *dsx*, and *ppk* *GAL4* lines, this is not observed and they all express strongly. Consistent with a requirement for Nup54 early in development, *dsx* is expressed earlier than *fru* and *ppk*. Differential effects on receptivity, but not egg-laying by *dsx* driven rescue by *Nup54*, however, more likely indicates that receptivity and egg-laying are governed by different neuronal circuits. These results are supported by previous observations that G(o) is not required in *fru*, but needed in *dsx* and *ppk* neurons to reduce receptivity [21]. Likewise, membrane-tethered SP can only induce oviposition in the absence of SPR in *dsx*, but not *fru* and *ppk* neurons [21].

### Roles of *Nups* in speciation

New species can arise through the selection of new features enhancing the display of sexual attributes and altered courtship behavior. A driving force to speciation can be sexual conflict imposed by male-directed post-mating responses by females [22–24]. In *D. melanogaster*, females respond to male-derived SP by reducing receptivity and increasing egg-laying, but need to adapt their physiological status to environmental conditions. Thus, the possibility to limit the male influence imposed by SP is in the female interest of optimizing reproductive success when resources to produce eggs are scarce. In this context, *Nup54* regulation could impact on egg-laying in dedicated circuits such as the brain *ppk* neurons. Consistent with this interpretation, the *Nup54* promoter region has undergone a dramatic change compared to closely related species, while no adaptive changes were detected in the protein-coding part. The QB62 deletion in the promoter region of *Nup54* affected the ELAV distribution of *ppk*-expressing neurons and such deletion mimics the wild-type allele condition in sister species of *D. melanogaster*. While we did not perform a direct interspecies allele swap, our result suggests that differences in regulation of *Nup54* might be critical in mediating interspecies differences in female reproductive behavior. How allelic variants of Nups lead to reproductive advantages, however, needs to be further explored, but could involve subtle changes in neuronal wiring that alters behavioral preference. In addition, since *Nup54* is required in all cells, varying its concentration could have profound effects on the general expression of genes and select for a specific compensatory genetic element from the mating partner to prevent deleterious effects on the fitness of the progeny [25–27], but whether nucleo-cytoplasmic shuttling is affected in species with a QB62-like promoter remains to be tested. The regulation of gene expression at the nuclear pore has been linked to speciation, because essential *Nup96* and *Nup160* have been identified in causing hybrid sterility or lethality. Intriguingly, Nup96 and Nup160 are part of the larger Nup107 subcomplex and four out of eight proteins from this complex are under adaptive selection in *D. simulans* [27].

*Nup54* has also been found to interact with the piRNA pathway and interspecific hybrids phenocopy piRNA pathway mutants [48–50]. The piRNA pathway is involved in suppressing the mobilization of transposons in the germline. PIWI further interacts with ELYS at nuclear pores indicating that RNA export through nuclear pores is critical for transposon suppression [51, 52]. In particular, channel Nups are required for piRNA biogenesis of the *flamenco* locus and through transposon silencing can impact on fecundity [46]. The driving forces behind the role of Nups in speciation clearly seem to go beyond hybrid sterility or lethality. Hence, the molecular mechanism leading to speciation through Nups needs to be further explored.

## Conclusions

We have identified eight *ppk*-expressing neurons in the brain as a cellular focus preventing female escape from male manipulation. Changes in neuronal wiring likely directed in response to sexual conflict arising from male-derived SP to direct the female post-mating response marks an early event in the splitting of species and links differentiation of key neurons involved in female control of the reproduction to fitness as a result of sexual conflict. Our results indicate a central role for the nuclear pore in implementing alterations of gene expression impacting on neuronal wiring, which might have been critical at the onset of speciation.

## Methods

Flies were kept on standard cornmeal-agar food (1%industrial-grade agar, 2.1% dried yeast, 8.6% dextrose, 9.7% cornmeal, and 0.25% Nipagin, all in (w/v)) in a 12-h light: 12-h dark cycle. Sexually mature 3–5-day-old virgin females were injected and examined for their post-mating behaviors non-blinded as described previously [4, 15]. For injections, virgin female flies were cooled to 4°C and 3 pmol SP in 50 nl Ringer’s solution was injected. The ovaries were analyzed as previously described [4].

In a genetic EMS screen for oogenesis mutants, one class was identified that had normal oogenesis, but females did not lay eggs [30]. To test whether in this class of mutants, we can identify ones that are insensitive to SP, we first validated that homozygous females did not increase egg-laying upon SP injection. Then, we tested whether homozygous females injected with SP reduce receptivity.

For the analysis of larval and adult axonal projection expressing green fluorescent protein (GFP), tissues were dissected in PBS, fixed in 4% paraformaldehyde in PBS for 15 min, washed in PBST (PBS with BSA and 0.3% Triton-x 100). Aberrant projections in the larval ventral nerve cord were classified as mild, if there were slight mis-projections in the anterior region, as moderate, if connectives or commissures were thinned indicative of absent projections and as severe, if one or more connectives or commissures were completely disrupted.

Aberrant projections in the adult brain were classified as mild, if there were slight mis-projections, as moderate, if some projections did not innervate the target area and as severe, if entire projections were missing.

In situ antibody stainings were done as described previously [31] using rat anti-HA (MAb 3F10, 1:20; Roche) and mouse anti-ELAV (MAb 7D, 1:20, which recognizes 7 amino acids unique to ELAV) [53] and visualization with Alexa Fluor 488 or Alexa Fluor 647-coupled secondary antibodies (1:250; Molecular Probes or Invitrogen, A11034). DAPI (4’,6-diamidino-2-phenylindole) was used at 1 μg/ml. For imaging, the tissues were mounted in Vectashield (Vector Labs) and visualized with confocal microscopy using a Leica TCS SP8. Images were processed using Fiji. Pictures of fly parts were taken with a Zeiss Stemi200-CS and Zen Blue software. Since egg-laying and receptivity data are skewed, we used non-parametric Welch’s ANOVA followed by planned pairwise comparisons with Dunnett’s multiple comparison test done for statistical analysis using Graphpad prism.

The *UAS* construct with a N-terminally HA-tagged *Nup54* was generated in a three-way ligation with fragments of *Nup54* amplified from cDNA by RT-PCR that were cloned into a modified *pUC*, *pUC 3GLA UAS HA*, with NheI and Acc65I with a blunt site in between. The 5′ part was amplified using primers 8831F1 (CATCGCTAGCGCCTGCAGGATCGTTCTTCGGATCCAACACGTCGCTGG) and 8831R1 (CTGAGAGTTCTCTGCAGAAGTTAAGAGCCAC), and the 3′ part with primers 8831F2 (CTGTCAAGCCACACCAGCAACAAGTGATTC) and 8831R2 (GACAGGTACCTATCACGATTGTCGCAGCTCGGGCAGTC) by PCR with Pwo (Roche) and sequenced. The genomic rescue construct was generated in a three-way ligation by cloning the promoter fragment amplified from genomic DNA with primers 8831F1g (GTGGAATTCCGGAGGCCACTAGAACATATACTTGTC) and 8831R1g (GGCGTGCTTGTTGCTCCCAGCGACGTGTTG) and the cDNA part amplified from the *UAS* construct with primers 8831F3 (GGCCAAAACAACCGGTGGCCTCTTCGGATC) and 8831R3 (GGCGTGCTTGTTGCTCCCAGCGACGTGTTGTAGCTCGAGGATTGTCGCAGCTCGGGCAGTC) using EcoRI and XhoI sites into a modified *pUC*, *pUC 3GLA* that adds a C-terminal HA tag and sequenced [54]. Swapping the *Nup54* wild type promoter was done by digesting the parent plasmid with BspEI and NgoMIV to insert the *Nup54*^*QB62*^ promoter or the promoter from *Drosophila simulans* (S-23, Ethiopie 225, Welo Ataye River, F. Leumeunier, gift from S. Collier Cambridge) from a PCR amplified with primers 8831F3BspE (GCTTAGGATCCGATCGCGTGGAATTCCGGAGGCCACTAGAACATATACTTGTC) and 8831promR (CGAAGAGGCCACCGGTTGTTTTGGCCGGCGTGCTTGTTGCTCCCAGCGACGGTTG). The resulting *pUC 3GLA gNup54*^*QB62*^ and *pUC 3GLA gNup54*^*Dsim*^ constructs were validated by sequencing with primer 8831promR2seq (GGCTGGTTGCAGCTGTGCCTCCAAAC). The *D. elegans* rescue construct was generated in a three-way ligation by replacing the part between NgoMIV and MfeI with the corresponding part amplified from *D. elegans* cDNA by RT-PCR with primers 8831Fele (CCAAAACAACCGGAGGCCTCTTCGGAAC) and 8831R1ele (CGTGGATCCGAAGGCTCCGCCCCCAAAGCCAGTG), and a BamHI/MfeI fragment obtained from the UAS construct. Transgenic flies were generated by phiC31-mediated transformation using landing site 76A (*PBac{y+ -attP-3B}VK00002*), where the GFP marker had been removed by Cre/Lox-mediated recombination. *Df(2R)9B4* was generated by FLP/FRT-mediated recombination between the two transposon insertion lines, *P{XP}CG8525*^*d06853*^ and *PBac{RB}DUBAI*^*e00699*^ as described [32]. The *tubGAL4* line was a third chromosomal insert (Bloomington #5138), and *UAS FRTGFPstopFRT TNT* and the other lines have been previously described [19, 21, 31].

Genomic DNA was extracted from *Nup54*^*QB62*^ as described [55]. The promoter fragment was amplified with primers 8831pF1 (GGATCTGGTGAGCAGAGGTTGCGATG) and 8831pR1 (GCCGCACAGTTTGGGTTGCCTTTC) and sequenced. Reverse transcription-quantitative polymerase chain reaction (RT-qPCR) was done by extracting total RNA using Tri-reagent (SIGMA), and RT was done with Superscript II (Invitrogen) according to the manufacturer’s instructions using an oligo dT primer. For qPCR, 1.5 μl cDNA with the SensiFAST SYBR No-Rox kit (Bioline) was used with primers Nup54qF1 (CTGCCACAGCGAAGATACT) and Nup54qR1 (CAGCATGTTCTGTAGCTTGGTGC), and ewg4F1 and ewg5R1 [56]. Amplification was done in a Applied Biosystems ABI Prism 7000 with a 3-min initial denaturation at 95°C followed by 40 cycles with a 15-s denaturation at 95°C and 60 s extension at 60°C. Quantification was done according to the ∆CT method as described [44].

Structural analysis was done by PyMol. Expression data were retrieved from FlyBase (flybase.org) [35, 36]. The *Nup54* ORF and extended gene sequences were retrieved from FlyBase (flybase.org) and aligned using muscle within MEGA [57]. *D. melanogaster* polymorphism data was retrieved from the Drosophila Genetic Reference Panel (http://dgrp2.gnets.ncsu.edu/) and used along with interspecies divergence data (*D. melanogaster – D. simulans*) to conduct McDonald Kreitman’s tests of selection for the ORF and extended gene region. Tests were conducted by comparing nonsynonymous to synonymous substitutions and polymorphisms (ORF) as well as noncoding upstream to silent (within gene synonymous and intron) substitutions and polymorphism [58].

We also tested for evidence of nonrandom accumulation of substitutions along the *Nup54* extended gene region. The method tests for significant deviations from a uniform distribution of substitutions using an empirical cumulative distribution function. The function (G) detects monotonic increases in substitutions (n) measured as the difference between the relative occurrence of a nucleotide change and its relative position in the alignment [59]. Whether differences between the values of the G function (ΔG) between substitutional events deviates from a random accumulation of changes is tested using Monte Carlo simulations to produce 100,000 samples of *n* events by sampling sites without replacement along the alignment [59].

## Supplementary Information

**Additional file 1 ***Nup54* is required before neuronal maturation for establishing the post-mating response. A) Receptivity of control (white for Ringer and black for SP injection) and transheterozygous *Nup54*^*MB03363*^*/Df(2R)9B4* expressing *UASNup54::HA* with *tubGAL4* (blue) ubiquitously, in neurons with *elavGAL4*^*C155*^ (purple) or glia with *repoGAL4* (yellow), and in *ppkGAL4* (orange), *fruGAL4* (green) and *dsxGAL4* (red) patterns after sex-peptide (SP, dark color) or Ringer’s (R, light color) injection measured by counting mating females in a 1 h time period 3 h after SP or R injection, respectively. Means with the standard error for three experiments with 15-21 females each are shown, and statistically significant differences from ANOVA post-hoc pairwise comparisons are indicated by different letters (p≤0.05). B) Oviposition of control (white for Ringer and black for SP injection) and transheterozygous *Nup54*^*MB03363*^*/Df(2R)9B4* expressing *UASNup54::HA* with *tubGAL4* (blue) ubiquitously, in neurons with *elavGAL4*^*C155*^ (purple) or glia with *repoGAL4* (yellow), and in *ppkGAL4* (orange), *fruGAL4* (green) and *dsxGAL4* (red) patterns after sex-peptide (SP, dark color) or Ringer’s (R, light color) injection shown as means of eggs laid in 18 h with the standard error for 10-15 females each, respectively, and statistically significant differences from ANOVA post-hoc pairwise comparisons are indicated by different letters (a, b: p≤0.001, c: ns). The data underlying the presented graphs are in Additional file 6.

**Additional file 2 **Gross organization of the brain and ventral nerve cord is normal in *Nup54* mutants. A-F) Projections of *dsx* neurons in the brain (A and D) and ventral nerve cord (VNC, B and E) and *dsx* expression in the genital tract (C and F) visualized by expression of membrane-bound CD8GFP from *UAS* by *dsxGAL4* show no gross alterations in *Df(2R)9B4 gNup54*^*QB62*^ (D-E) compared to wild type (A-C). st: spermathecae, sr: seminal receptaculum. The scale bar in D is 100 μm.

**Additional file 3 ***Nup54 RNAi* in *doublesex* expressing neurons reveals a separable sex-peptide response in receptivity and oviposition and a role in sexual differentiation. A) Receptivity after *Nup54 RNAi* knock-down from *UAS P{GD14041}v42153; P{GD14041}v42154* inserts in neurons with *elavGAL4*^*C155*^ (yellow) and in *ppkGAL4* (orange), *fruGAL4* (green) and *dsxGAL4* (red) patterns after sex-peptide (SP, dark color) or Ringer’s (R, light color) injection measured by counting mating females in a 1 h time period 3 h after SP or R injection, respectively. Means with the standard error for three experiments with 16 females each are shown, and statistically significant differences from ANOVA post-hoc pairwise comparisons are indicated by different letters (p≤0.001). B) Oviposition after *Nup54 RNAi* knock-down from *UAS P{GD14041}v42153; P{GD14041}v42154* inserts in neurons with *elavGAL4*^*C155*^ (yellow) and in *ppkGAL4* (orange), *fruGAL4* (green) and *dsxGAL4* (red) patterns after sex-peptide (SP, dark color) or Ringer’s (R, light color) injection shown as means of eggs laid in 18 h with the standard error for 8 females each, respectively, and statistically significant differences from ANOVA post-hoc pairwise comparisons are indicated by different letters (p≤0.0001). C, D) Genitals of control females (C) and females expressing *Nup62 RNAi* from *UAS* with *dsxGAL4* (D). The scale bar in C is 20 μm. E, F) Front legs of control males (E) and males expressing *Nup62 RNAi* from *UAS* with *dsxGAL4* (F). Arrowheads indicate the position of sex combs. The scale bar in E is 100 μm. The data underlying the presented graphs are in Additional file 6.

**Additional file 4 **Expression of *Nup54*, *Nup58* and *Nup62*. A-C) Profile of *Nup54*, *Nup58* and *Nup62* expression during development from RNAseq summarized from flybase. D-F) Profile of *Nup54*, *Nup58* and *Nup62* expression in various tissues from microarrays summarized from flybase. The data underlying the presented graphs are in Additional file 6.

**Additional file 5 **NUP54 is highly conserved, but variation in the FG repeat region is not the cause of an altered post-mating response. A) Sequence alignment of NUP54 from closely related species. Amino acids deviating from *D. melanogaster* are indicated in black. Intron positions are indicated by black arrowheads and the stop codon of the *Nup54*^*MB03363*^ allele is indicated by a red arrow head. Green and white filled arrowheads indicate maintained (>1%) and rare (<1%) polymorphisms with amino acid changes indicated on top. The line below the sequence indicates the NgoMIV-BamHI fragment that was replaced in the *gNup54elegans* construct. Nucleotides 1-115 according to human NUP54 impasse the FG region, nucleotides 116-346 the α/β region and nucleotides 346-494 the α-helical region. The amino acids in exon 3 have been shown to bind to NUP62 and the amino acids in exon 5 bind to NUP58. B) Receptivity of wild type, *gNup54* and *gNup54elegans* females homozygous for *Df(2R)9B4* after sex-peptide (SP) or Ringer’s (R) injection measured by counting mating females in a 1 hr time period 3 hr after SP or R injection, respectively. Means with the standard error for three experiments with 8-15 females each are shown, and statistically significant differences from ANOVA post-hoc pairwise comparisons are indicated by different letters (p≤0.001). C) Oviposition of *gNup54* and *gNup54elegans* females homozygous for *Df(2R)9B4* after sex-peptide (SP) or Ringer’s (R) injection shown as means of eggs laid in 18 h with the standard error for 10 females each, respectively. Statistically significant differences from ANOVA post-hoc pairwise comparisons are indicated by different letters (p≤0.001). The data underlying the presented graphs are in Additional file 6.

**Additional file 6.** Source data underlying the presented graphs.

## Data Availability

All data generated or analyzed during this study are included in this published article and its supplementary information files. Reagents are available from the corresponding author upon request.

## Abbreviations

Nup: Nuclear pore protein
SP: Sex-peptide
NPC: Nuclear pore complex
RNAi: Ribonucleic acid interference
SPR: Sex-peptide receptor
ppk: Pickpocket
fru: Fruitless
dsx: Doublesex
GFP: Green fluorescent protein
ORF: Open reading frame
TNT: Tetanus toxin
ELAV: Embryonic lethal abnormal visual system
