# Supplementary figures and images for: Channel nuclear pore protein 54 directs sexual differentiation and neuronal wiring of female reproductive behaviors in Drosophila
Source: BMC Biol. 2021 Oct 20;19:226. doi: 10.1186/s12915-021-01154-6 (PMC8527774; doi:10.1186/s12915-021-01154-6)

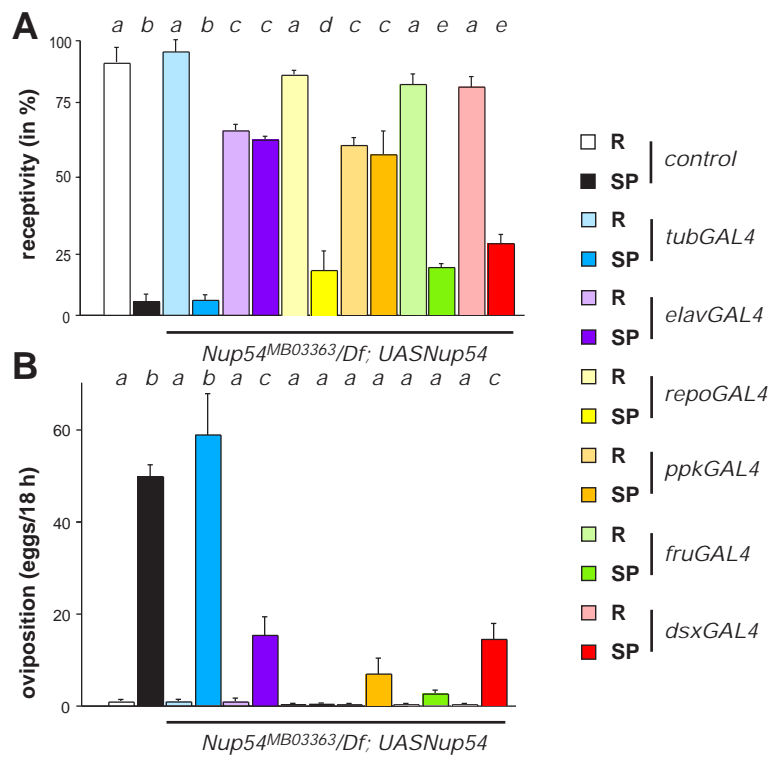

Supplement: Supplementary file 1 — Additional file 1 Nup54 is required before neuronal maturation for establishing the post-mating response. A) Receptivity of control (white for Ringer and black for SP injection) and transheterozygous Nup54MB03363/Df(2R)9B4 expressing UASNup54::HA with tubGAL4 (blue) ubiquitously, in neurons with elavGAL4C155 (purple) or glia with repoGAL4 (yellow), and in ppkGAL4 (orange), fruGAL4 (green) and dsxGAL4 (red) patterns after sex-peptide (SP, dark color) or Ringer’s (R, light color) injection measured by counting mating females in a 1 h time period 3 h after SP or R injection, respectively. Means with the standard error for three experiments with 15-21 females each are shown, and statistically significant differences from ANOVA post-hoc pairwise comparisons are indicated by different letters (p≤0.05). B) Oviposition of control (white for Ringer and black for SP injection) and transheterozygous Nup54MB03363/Df(2R)9B4 expressing UASNup54::HA with tubGAL4 (blue) ubiquitously, in neurons with elavGAL4C155 (purple) or glia with repoGAL4 (yellow), and in ppkGAL4 (orange), fruGAL4 (green) and dsxGAL4 (red) patterns after sex-peptide (SP, dark color) or Ringer’s (R, light color) injection shown as means of eggs laid in 18 h with the standard error for 10-15 females each, respectively, and statistically significant differences from ANOVA post-hoc pairwise comparisons are indicated by different letters (a, b: p≤0.001, c: ns). The data underlying the presented graphs are in Additional file 6. [file 12915_2021_1154_MOESM1_ESM.pdf]

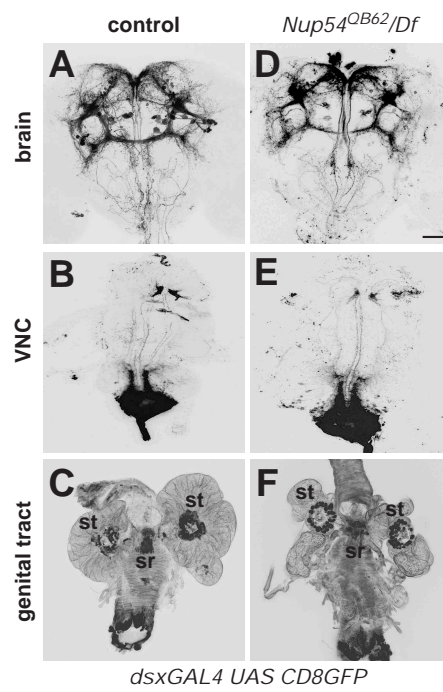

Supplement: Supplementary file 2 — Additional file 2 Gross organization of the brain and ventral nerve cord is normal in Nup54 mutants. A-F) Projections of dsx neurons in the brain (A and D) and ventral nerve cord (VNC, B and E) and dsx expression in the genital tract (C and F) visualized by expression of membrane-bound CD8GFP from UAS by dsxGAL4 show no gross alterations in Df(2R)9B4 gNup54QB62 (D-E) compared to wild type (A-C). st: spermathecae, sr: seminal receptaculum. The scale bar in D is 100 μm. [file 12915_2021_1154_MOESM2_ESM.pdf]

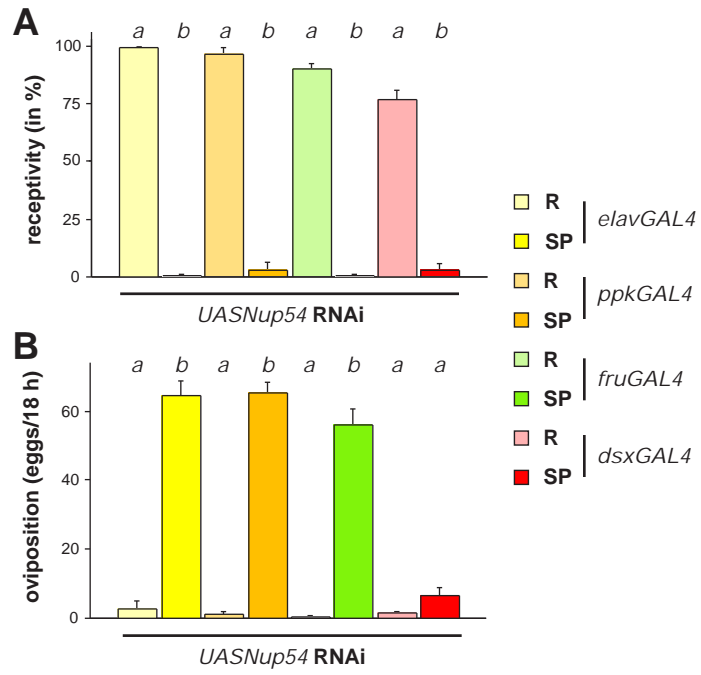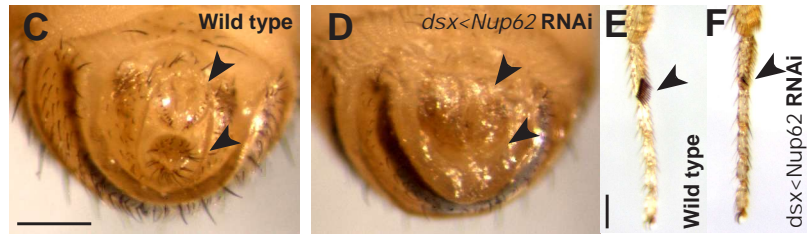

Supplement: Supplementary file 3 — Additional file 3 Nup54 RNAi in doublesex expressing neurons reveals a separable sex-peptide response in receptivity and oviposition and a role in sexual differentiation. A) Receptivity after Nup54 RNAi knock-down from UAS P{GD14041}v42153; P{GD14041}v42154 inserts in neurons with elavGAL4C155 (yellow) and in ppkGAL4 (orange), fruGAL4 (green) and dsxGAL4 (red) patterns after sex-peptide (SP, dark color) or Ringer’s (R, light color) injection measured by counting mating females in a 1 h time period 3 h after SP or R injection, respectively. Means with the standard error for three experiments with 16 females each are shown, and statistically significant differences from ANOVA post-hoc pairwise comparisons are indicated by different letters (p≤0.001). B) Oviposition after Nup54 RNAi knock-down from UAS P{GD14041}v42153; P{GD14041}v42154 inserts in neurons with elavGAL4C155 (yellow) and in ppkGAL4 (orange), fruGAL4 (green) and dsxGAL4 (red) patterns after sex-peptide (SP, dark color) or Ringer’s (R, light color) injection shown as means of eggs laid in 18 h with the standard error for 8 females each, respectively, and statistically significant differences from ANOVA post-hoc pairwise comparisons are indicated by different letters (p≤0.0001). C, D) Genitals of control females (C) and females expressing Nup62 RNAi from UAS with dsxGAL4 (D). The scale bar in C is 20 μm. E, F) Front legs of control males (E) and males expressing Nup62 RNAi from UAS with dsxGAL4 (F). Arrowheads indicate the position of sex combs. The scale bar in E is 100 μm. The data underlying the presented graphs are in Additional file 6. [file 12915_2021_1154_MOESM3_ESM.pdf]

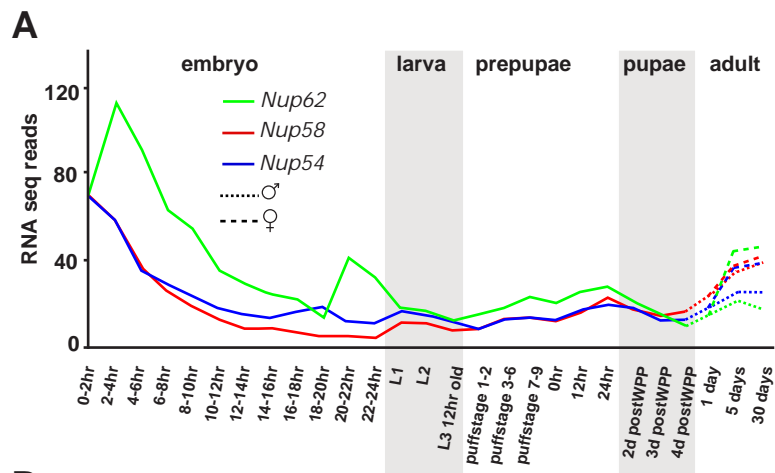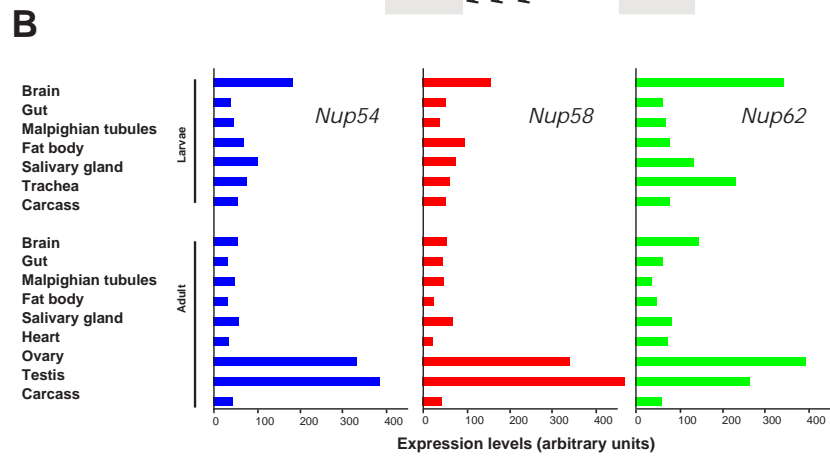

Supplement: Supplementary file 4 — Additional file 4 Expression of Nup54, Nup58 and Nup62. A-C) Profile of Nup54, Nup58 and Nup62 expression during development from RNAseq summarized from flybase. D-F) Profile of Nup54, Nup58 and Nup62 expression in various tissues from microarrays summarized from flybase. The data underlying the presented graphs are in Additional file 6. [file 12915_2021_1154_MOESM4_ESM.pdf]

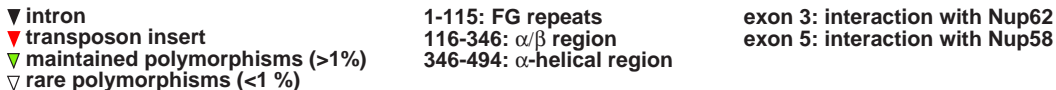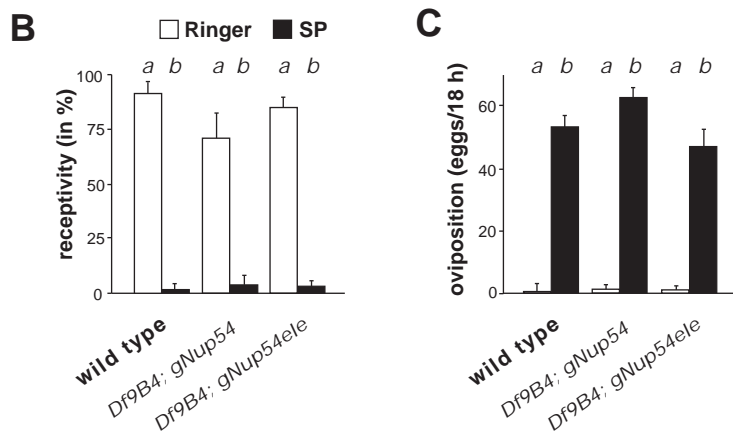

Supplement: Supplementary file 5 — Additional file 5 NUP54 is highly conserved, but variation in the FG repeat region is not the cause of an altered post-mating response. A) Sequence alignment of NUP54 from closely related species. Amino acids deviating from D. melanogaster are indicated in black. Intron positions are indicated by black arrowheads and the stop codon of the Nup54MB03363 allele is indicated by a red arrow head. Green and white filled arrowheads indicate maintained (>1%) and rare (<1%) polymorphisms with amino acid changes indicated on top. The line below the sequence indicates the NgoMIV-BamHI fragment that was replaced in the gNup54elegans construct. Nucleotides 1-115 according to human NUP54 impasse the FG region, nucleotides 116-346 the α/β region and nucleotides 346-494 the α-helical region. The amino acids in exon 3 have been shown to bind to NUP62 and the amino acids in exon 5 bind to NUP58. B) Receptivity of wild type, gNup54 and gNup54elegans females homozygous for Df(2R)9B4 after sex-peptide (SP) or Ringer’s (R) injection measured by counting mating females in a 1 hr time period 3 hr after SP or R injection, respectively. Means with the standard error for three experiments with 8-15 females each are shown, and statistically significant differences from ANOVA post-hoc pairwise comparisons are indicated by different letters (p≤0.001). C) Oviposition of gNup54 and gNup54elegans females homozygous for Df(2R)9B4 after sex-peptide (SP) or Ringer’s (R) injection shown as means of eggs laid in 18 h with the standard error for 10 females each, respectively. Statistically significant differences from ANOVA post-hoc pairwise comparisons are indicated by different letters (p≤0.001). The data underlying the presented graphs are in Additional file 6. [file 12915_2021_1154_MOESM5_ESM.pdf]
